# Hidden Targets in RAF Signalling Pathways to Block Oncogenic RAS Signalling

**DOI:** 10.3390/genes12040553

**Published:** 2021-04-10

**Authors:** Aoife A. Nolan, Nourhan K. Aboud, Walter Kolch, David Matallanas

**Affiliations:** 1 Systems Biology Ireland, School of Medicine, University College Dublin, Belfield, Dublin 4, Ireland; aoife.a.nolan@ucdconnect.ie (A.A.N.); nourhan.aboud@ucdconnect.ie (N.K.A.); 2 Conway Institute of Biomolecular & Biomedical Research, University College Dublin, Belfield, Dublin 4, Ireland

**Keywords:** RAF kinase-independent, RAS, MST2, ASK, PLK, RHO-α, apoptosis, cell cycle, cancer therapy

## Abstract

Oncogenic RAS (Rat sarcoma) mutations drive more than half of human cancers, and RAS inhibition is the holy grail of oncology. Thirty years of relentless efforts and harsh disappointments have taught us about the intricacies of oncogenic RAS signalling that allow us to now get a pharmacological grip on this elusive protein. The inhibition of effector pathways, such as the RAF-MEK-ERK pathway, has largely proven disappointing. Thus far, most of these efforts were aimed at blocking the activation of ERK. Here, we discuss RAF-dependent pathways that are regulated through RAF functions independent of catalytic activity and their potential role as targets to block oncogenic RAS signalling. We focus on the now well documented roles of RAF kinase-independent functions in apoptosis, cell cycle progression and cell migration.

## 1. Introduction

RAS (Rat sarcoma) proteins are mutated in ca. 20% of all human cancers, with prevalent and deadly cancers such as colorectal, lung and pancreatic cancer featuring 40%, 20–40%, and >90% RAS mutations [1]. RAS proteins often have been described as molecular switches that cycle between GDP-bound off and GTP-bound on states. When switched on by the (normally receptor-induced) exchange of GDP versus GTP, they can bind to an array of >20 different types of effector proteins which mediate the downstream biochemical and biological effects of RAS [2,3]. Oncogenic mutations keep RAS proteins in the GTP bound state resulting in the constitutive activation of pathways that stimulate cell proliferation, survival, invasiveness and drug resistance. Thus, inhibiting RAS has been an early aim for the development of targeted therapies for cancer [4]. 

**Figure 1:**
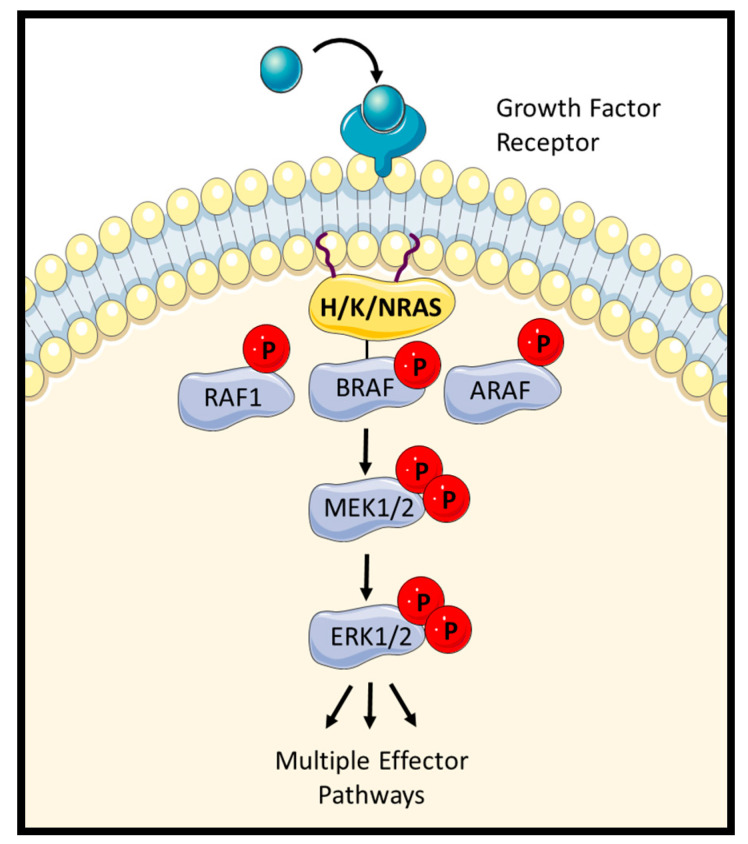
The RAF-MEK-ERK pathway is activated by H/K and NRAS upon extracellular stimuli. ERK1/2 phosphorylate over 50 substrates and control different cell fate.

When efforts to inhibit RAS directly failed, the attention turned to the inhibition of downstream effector pathways. A main effector of oncogenic RAS signalling is the RAF-MEK-ERK pathway (Figure 1). This pathway is a cascade of three kinases. The first, RAF (Rapid Accelerated Fibrosarcoma), binds to GTP-loaded RAS and is a direct RAS effector. RAS-activated RAF phosphorylates and activates MEK (Dual specificity mitogen-activated protein kinase kinase 1), which in turn phosphorylates and activates ERK (extracellular signal-regulated kinase) [5]. RAF is a family of three kinases: RAF1, BRAF, and ARAF. The RAF-MEK-ERK pathway drives several features of oncogenic transformation, and BRAF is an oncogene in its own right that is frequently mutated in melanoma, colorectal cancer, and lung cancer, amongst others [5,6]. Thus, drugs targeting the RAF-MEK-ERK pathway seemed a logical and promising strategy. Potent RAF and MEK inhibitors were developed and several are used in the clinic [6]. However, while effective against some mutant BRAF-driven cancers, such as melanoma, they proved ineffective against RAS-mutated cancers [4,6]. A main reason is that RAS induces homo- and heterodimerization of RAF kinases, and that the dimer is resistant to drug inhibition. The drug resistance is caused by the ability of a drug-bound, catalytically inhibited RAF protomer to allosterically transactivate the kinase activity of the other protomer [7,8]. This mechanism leads to a paradoxical activation of ERK in RAS-mutated cells in response to RAF inhibitors [9,10,11]. We have recently shown that this impasse can be broken by combining RAF inhibitors, chosen by a sophisticated computational model, that will effectively block ERK activation in mutant RAS cells [12]. Other pharmacological approaches to overcome RAF dimer-induced resistance to RAF inhibitors are being pursued as well [13,14,15]. All these approaches focus on preventing the reactivation of ERK signalling.

In this review, we focus on a complementary arm of RAF functions, which is the control of signalling pathways independent of RAF kinase catalytic functions. RAF kinases have several kinase-independent functions, which are relevant for cancer development and progression. Here, we summarize how these kinase-independent RAF functions contribute to cancer and explore how they could be targeted.

## 2. RAF Interacting Proteins Regulated in a Kinase-Independent Fashion

The only commonly accepted substrates for RAF kinases are the MEK1/2 kinases, whose only known substrates are ERK1/2. Activation of this pathway through oncogenic mutations of RAS, RAF or MEK kinases can drive cancer cell proliferation. However, several lines of evidence now strongly support a contribution of kinase - independent functions to the oncogenic capacity of this pathway. This was first realized in 2001, when reports were published that knocking out the *RAF1* gene in mice causes apoptosis independently of its kinase function [16,17]. Mikula et al. showed that knocking out *RAF1* had no impact on the activation of the ERK pathway but resulted in increased apoptosis mainly in the liver and haematopoietic system [17]. Later in the same year, these publications were followed by a report showing that RAF1 counteracts apoptosis by binding to and inhibiting the proapoptotic kinase ASK1 without the need for RAF1 catalytic activity [18]. Hüser et al. also showed that knocking out *RAF1* increased apoptosis in embryonal tissues without affecting ERK activation, and that expression of a RAF1 mutant which cannot be activated could rescue the apoptosis defect [16]. These results strongly suggested that RAF1 counteracts apoptosis independent of its ability to activate the ERK pathway and may be independent of its catalytic activity altogether. However, a mechanism was lacking. Since then, several proteins have been described as being regulated by RAF kinases independently of RAF kinase activity. These proteins can be grouped by the biological functions that they mediate which include cell death, cell cycle regulation and migration. In this section, we discuss them in relation to their main function. 

### 2.1. RAF Proteins Inhibiting Cell Death in a Kinase-Independent Manner

The kinase-independent role of RAF1 as a negative regulator of apoptosis is well characterized, and here, we review how RAF1 regulates the three effector proteins identified so far—ASK1, MST2 and BAD. 

#### 2.1.1. Apoptosis Signal-Regulating Kinase 1 (ASK1) and the Stress MAPK Pathways

ASK1 (also known as MAP3K5) is a MAPKKK that has been shown to trigger apoptosis in response to oxidative stress [19]. ASK1 is a serine/threonine kinase that can activate the stress-induced MAPK pathways, JNK (c-Jun N-terminal kinases) and p38 [20]. In 2001, Fu’s group showed that RAF1 overexpression inhibits ASK1 proapoptotic signalling [18]. Importantly, this work showed that ASK1-dependent apoptosis was inhibited by RAF1 independent of its canonical effectors MEK1/2 and ERK1/2. ASK1 and RAF1 were shown to interact in co-immunoprecipitations assays, and further biochemical characterization mapped the protein domains involved in the ASK1–RAF1 interaction. Both wildtype RAF1 and kinase-defective RAF1 mutants bind to the N-terminal regulatory domain of ASK1 and inhibit its activation. This is probably the first confirmation of a RAF kinase-independent function. 

**Figure 2:**
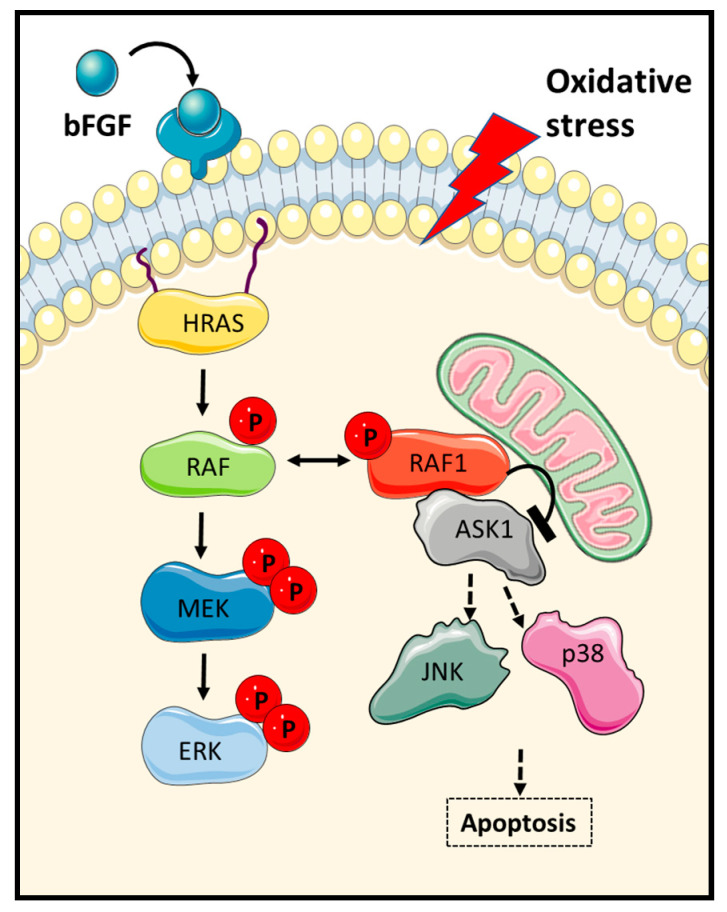
RAF1 kinase-independent regulation of ASK1 proapoptotic signalling. FGF activation promotes RAF1–ASK1 complex localization in the mitochondria. Oxidative stress prevents the inhibitory binding of RAF1 to ASK1 and leads to activation of stress MAPK.

**Figure 3:**
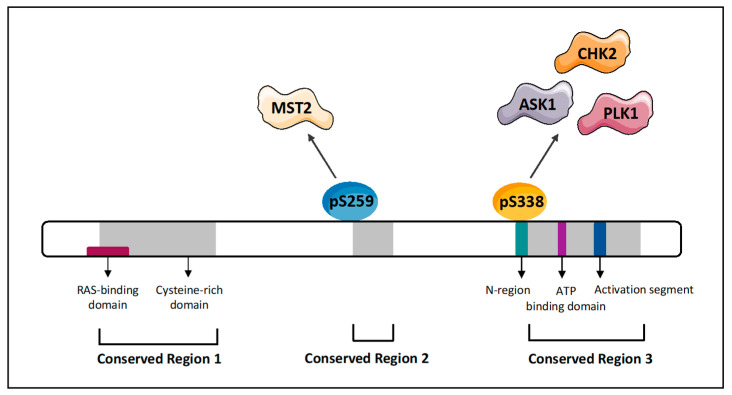
RAF1 protein structure. The phosphorylation sites indicate the residues that are known to be phosphorylated when RAF1 binds to its kinase-independent effectors.

The signalling pathway regulating the RAF1-ASK1 signal has been further mapped using both in vitro and in vivo experiments (Figure 2). Yamaguchi et al. showed that mice with cardiac muscle-specific ablation of the *RAF1* gene exhibit cardiac dysfunction caused by increased apoptosis of cardiomyocytes irrespective of MEK1/2 and ERK1/2 activity [21]. This work showed that loss of RAF1 expression caused an activation of ASK1 which was accompanied by the activation of JNK and p38. Importantly, knockout of the *ASK1* gene rescued the effect of *RAF1* deletion, genetically confirming that ASK1 mediates this mutant phenotype. These animals also showed a reduction in JNK and p38 activation, suggesting that these kinases are mediating the proapoptotic signal initiated by ASK1. Subsequent work confirmed that the JNK1 and p38 pathways are regulated by RAF1 through the modulation of ASK1 activation and provided more mechanistic insights for how this kinase-independent function of RAF1 works. Cheresh’s group showed that the negative regulation of ASK1 by RAF1 is related to the phosphorylation status of RAF1 [22]. They confirmed the RAF1 kinase-independent regulation of ASK1 and identified phosphorylation of the activating RAF1 residue Ser338 as a necessary step to mediate the interaction of RAF1 with ASK1 (Figure 3). Phosphorylation of this RAF1 residue is mediated by bFGF (fibroblast growth factor) in endothelial cells and prevents the activation of apoptosis by genotoxic agents. This work also showed that the activation of FGF receptor induce the increase in interaction between RAF1 and ASK1 in the mitochondria. Importantly, the interaction between RAF1 and ASK1 was shown to be regulated by HRAS preventing the activation of the p38 MAPK pathway in an ERK- and PI3K- (Phosphoinositide 3-kinase) independent fashion [23]. This work also indicated that the oncogenic HRAS^V12^ mutant exacerbated the inhibitory effect of HRAS on the ASK1 proapoptotic signal, while the dominant negative HRAS^N17^ mutant had no effect. These results indicate that ASK1 functions are regulated, at least in part, by HRAS through its main effector RAF1. 

#### 2.1.2. Mammalian STE20-Like Kinase 2 (MST2) and the Proapoptotic Hippo Pathway

The observations that ablation of *RAF1* caused widespread apoptosis and embryonic lethality, and that this was likely to be mediated by a kinase-independent function [16,17], led us to focus our attention on the mapping of the apoptotic mechanisms that were regulated by this kinase. To this end, we performed a proteomic screening using RAF1 as a bait. This experiment led to the identification of the kinase MST2 (also known as STK3) as a RAF1 interactor in COS-1 cells [24]. This interaction was detected when the cells were serum-deprived and reduced in growth factor stimulated cells, and MST2 also interacted with kinase-dead RAF1. 

*MST2* was originally cloned by Chernoff’s group as a close homologue of *MST1* [25], and MST1 was implicated as an effector in mediating proapoptotic RAS signalling [26]. MST1/2 activation requires homo- or heterodimerization and autophosphorylation of Thr180 (181 for MST1) in the activation loop [27]. O’Neill et al. showed that RAF1, but not wildtype BRAF, can bind to and inhibit MST2 kinase activation and subsequent MST2-mediated apoptosis through a two-pronged mechanism. First, RAF1 binding prevents MST1/2 dimerization necessary for activation. Second, RAF1 recruits a phosphatase that prevents the phosphorylation of MST2 on the activating Thr180. Neither mechanism requires RAF1 kinase activity. Proapoptotic signals induce the release of MST2 from RAF1 inhibitory binding and the activation of caspase-dependent apoptosis. Importantly, downregulation of MST2 in *RAF1* knock-out murine embryonic fibroblasts (MEFs) desensitised these cells to apoptosis signals [24], providing genetic evidence that RAF1 is a physiological antagonist of MST2-mediated apoptosis. 

RAF1 binds to the SARAH domain in MST2 [28]. The SARAH domain also mediates MST1/2 homo- and heterodimerization, explaining how RAF1 can disrupt MST1/2 dimers, MST2 activation, and binding of MST2 to its substrate LATS1 [28]. Vice versa, MST2 binds to residues 151 and 303 in RAF1, which overlap with the RAS- and the MEK-binding domains [24,29]. Therefore, not surprisingly, MST2 impedes the interaction of RAF1 with RAS and MEK and thereby inhibits the activation of ERK signalling. As a result, RAF1 and MST2 mutually inhibit each other. Interestingly, activation of MST2 induces phosphorylation of RAF1 at Ser259, which prevents RAF1 activation [30], but promotes the interaction with MST2. Thus, RAF1 that is inactive as MEK kinase is active as MST2 inhibitor. This mutual competition for MST2 and MEK1/2 binding to RAF-1 combined with changing affinities caused by phosphorylation gives rise to switchlike transitions that either enable cell proliferation through the RAF1 kinase-dependent stimulation of the ERK pathway or prevent apoptosis through the RAF1 kinase-independent inhibition of MST2. Interestingly, RAF1 phosphorylated on Ser259 is devoid of Ser338 phosphorylation [30], which is necessary for RAF1 binding to ASK1 [22], suggesting that RAF1 can inhibit apoptosis in two different activation states, i.e., by binding MST2 when inactive and by binding ASK1 (also PLK1 and CHK2 as explained below) when activated (Figure 3). It will be interesting to investigate the physiological role and molecular mechanisms of this coordination. 

**Figure 4:**
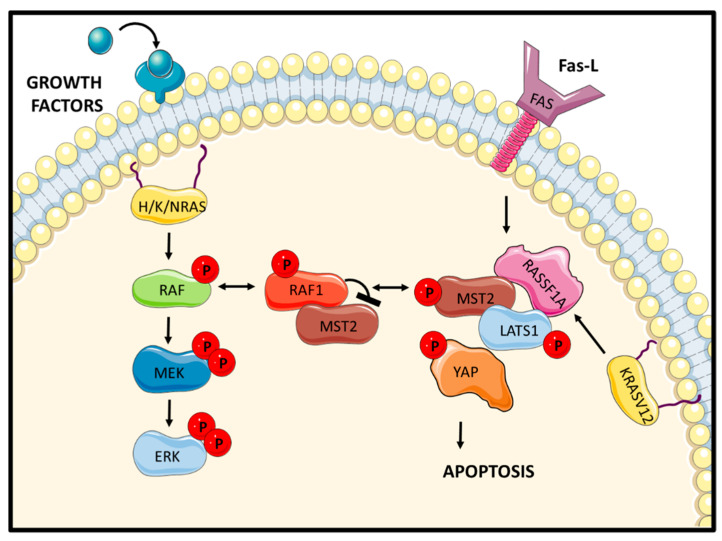
RAF1 negatively regulates the proapoptotic Hippo pathway by binding to MST2 upon growth factor stimulation. RASSF1A rescues MST2 from the inhibitory binding of RAF1 and regulates the activation of the core proteins of the Hippo pathway upon death receptor activation. Oncogenic KRAS also promotes the activation of the proapoptotic Hippo pathway while wildtype RAS isoforms promote RAF1–MST2 interaction.

Extensive work from our group using a combination of interaction proteomics experiments with molecular and functional experiments allowed us to map the signalling pathway that is activated by MST2 upon release from RAF1 inhibitory binding (Figure 4). This led to the identification of what now is known as the mammalian Hippo pathway [28,31,32] and has established this pathway together with ASK1 signalling as the main proapoptotic effectors of RAF proteins (for an extended review, see [33]). Briefly, we showed that the scaffold protein RASSF1A competes for RAF1 interaction with MST2 in response to proapoptotic signals releasing MST2 from RAF1 inhibition. MST2 then binds to RASSF1A (Ras association domain-containing protein 1A), dimerizes, becomes activated, and subsequently binds to and phosphorylates its substrate LATS1 (Large Tumour Suppressor 1). Activated LATS1 binds to and regulates different apoptotic effectors. LATS1 is a kinase but, similar to RAF1, also carries out important functions independent of its catalytic activity [34,35]. Our initial studies showed that it binds and regulates the co-transcription factor YAP1 (Yes-associated protein 1) and promotes YAP1 binding to the transcription factor p73 [36,37,38]. The YAP1–p73 complex induces the transcription of proapoptotic proteins, such as PUMA which ultimately leads to the activation of programmed cell death [28]. Further work revealed a second pathway that is stimulated by oncogenic KRAS [31]. KRAS is the only RAS family member that, in addition to stimulating cell transformation, can also induce apoptosis [39]. Mutated KRAS can bind RASSF1A and trigger activation of the proapoptotic MST2-LATS1 pathway. In this scenario, however, LATS1 induces the stabilization of the p53 tumour suppressor protein by sequestering MDM2 (Mouse double minute 2 homolog), a ubiquitin ligase that induces p53 degradation. Thus, MST2 can utilize at least two effector pathways for promoting apoptosis, one via p73 and another via p53. 

Further work revealed that the relation of the members of the RAF family and the Hippo pathway is rather extensive. The region that binds MST2 contains domains that diverge between the three RAF family members (ARAF, RAF1, BRAF) suggesting different affinities for MST2. This indeed was observed. Intriguingly, ARAF which has the lowest catalytic activity binds best to MST2, while BRAF which has the highest kinase activity does not bind MST2 [24,40,41]. ARAF regulates the function of MST2 during epithelial differentiation pointing to a specialised role of this interaction [41]. As already mentioned, BRAF, which has the highest kinase activity, did not interact with MST1/2 [24]. However, later work revealed that the oncogenic BRAF^V600E^ mutant can also bind and inhibit MST1 proapoptotic activation [42]. This suggests that inhibition of MST1/2 proapoptotic signalling is part of the mechanism of how BRAF^V600E^ induces cell transformation. 

Unfortunately, this RAF isoform specificity of MST2 regulation has contributed to the role of RAF kinases in MST2 regulation being depicted as controversial or being ignored altogether [43,44]. The Hippo/MST field developed from genetic studies of developmental pathways in the fruit fly *Drosophila melanogaster* [43], which only has one RAF gene corresponding to mammalian BRAF. Unsurprisingly, genetic interaction studies between RAF and Hippo in this organism came up empty handed, sometimes jumping to the categorical conclusion that these pathways cannot interact in mammals because they do not interact in flies [45,46,47]. Fortunately, the dogmatic dust around these controversies has settled now and given way to a clearer picture. The physiological relevance for the RAF1–MST1/2 interaction was demonstrated by experiments with animal models and clinical evidence. We showed that disruption of the RAF1–MST2 complex in zebrafish embryos results in an enlargement of the heart [29], confirming the important role that the MST2 pathway has in heart development [48]. Data from colorectal patients showed a significant inverse correlation between expression of MST2 and mutant KRAS, and in the few instances where these proteins were co-expressed, the tumours had high apoptosis rates. Intriguingly, MST2 expression was lost as tumours progressed to metastatic stages [31]. Importantly, work from Zhou’s group showed that NF2 (Neurofibromatosis 2), a member of the canonical hippo pathway, regulates the interaction between MST1/2 and RAF1 in mice liver downstream of FGFR4 (Fibroblast growth factor receptor 4) to regulate organ size, which is one of the best-known functions of the canonical Hippo pathway [49]. Finally, recent work from Barbacid’s group that will be discussed below shows that MST2 is one of the key mediators of the apoptosis caused by RAF1 ablation in murine *KRAS/p53* mutant lung tumours [50]. The emerging picture firmly places the RAF1–MST1/2 complex as a hub that coordinates apoptotic with developmental and oncogenic signalling. 

#### 2.1.3. RAF1 and BRAF Scaffolding Function Assisting the Inactivation of BAD

**Figure 5:**
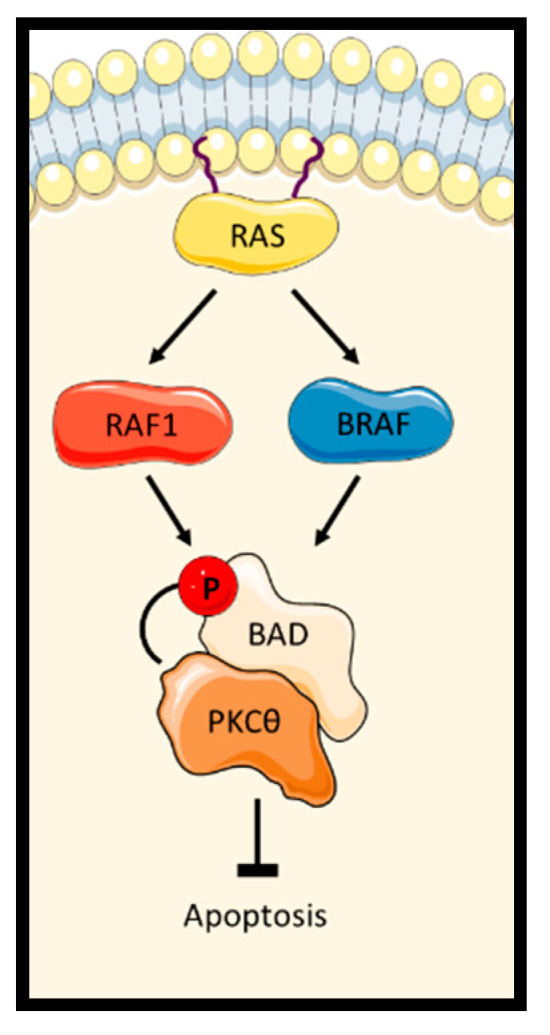
RAF1 and BRAF regulate the activation of apoptosis by modulating PKCθ phosphorylation of BAD.

BAD (Bcl-2 agonist of cell death) is a BH3-only member of the BCL2 family which can cause apoptosis by binding to and neutralizing the pro-survival effect of BCL2 proteins [51]. This function of BAD is regulated by phosphorylation. Although RAF1 was reported to promote survival by inactivating BAD through direct phosphorylation [52], subsequent results showed that BAD is not a credible RAF1 substrate [53]. This controversy was resolved later when it was discovered that RAF1 serves as an adaptor protein that promotes BAD binding to protein kinase-theta (PKCθ) downstream of RAS, which phosphorylates and inactivates BAD [54]. In this scenario, RAF1 stimulated both PKCθ activation and acted as a scaffold protein that, in a kinase-independent way, facilitated the interaction between PKCθ and its substrate BAD (Figure 5). BRAF also could stimulate PKCθ-mediated BAD phosphorylation and inactivation. RAF1 and BRAF cooperated in this function, suggesting that a RAF heterodimer is not only an effective activator of the ERK pathway, but also an efficient inhibitor of apoptosis by targeting BAD to it inhibitory kinase PKCθ.

### 2.2. Raf Kinase-Independent Regulation of Migration 

**Figure 6:**
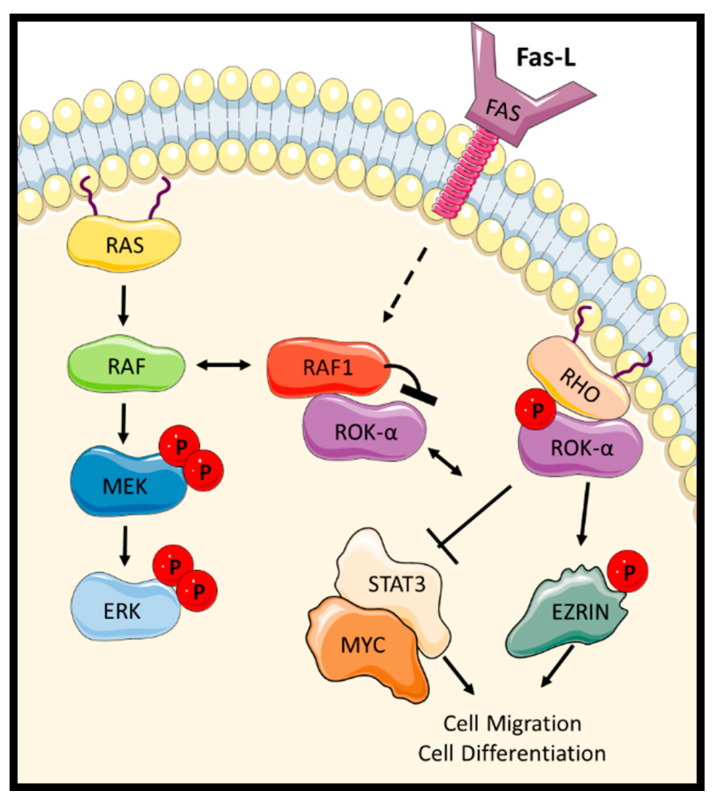
RAF1 interacts and inhibits the kinase activity of ROK-α. RHO binding to ROK-α rescues this kinase from the inhibitory effect of RAF1 and promotes the activation of cell migration and cell differentiation. FAS activation increases the formation of RAF1–ROK-α complex increasing apoptotic threshold.

The second function that was observed to be regulated by RAF1 in a kinase-independent fashion was migration through the modulation of RHO-dependent signalling. Conditional *RAF1* gene ablation in the skin of mice experiments indicated that the RHO effector ROK-α (active Rho Kinase) had a role in wound healing [55]. This work from Baccarini’s group also showed that *RAF1* deletion affected cell migration in cell lines such as keratinocytes and fibroblasts. Thus, *RAF1*-depleted cells showed a contracted phenotype and reduction in migration. Mechanistically, it was shown that *RAF1* deletion causes a hyperactivation of ROK-α and its mis-localisation to the plasma membrane, where ROK-α substrates are hyperphosphorylated leading to a collapse of the vimentin cytoskeleton and a constitutive contraction of cortical actin (Figure 6). RAF1 regulates ROK-α in a kinase-independent manner since overexpression of the kinase defective mutant RAF1 K375W was able to inhibit ROK-α and restore migration defects. Further work from this group showed that RAF1-mediated inhibition of ROK-α seems to be necessary for RAS-dependent tumorigenesis [56]. In particular, the formation of the RAF1–ROK-α complex in chemically induced murine skin carcinoma models allows the activation of STAT3 (Signal transducer and activator of transcription 3), and MYC (Myelocytomatosis) and cell de-differentiation. Importantly, *RAF1* ablation was sufficient to prevent RAS-dependent transformation in these animals. 

Subsequent work revealed an unusual molecular mechanism through which RAF1 inhibits ROK-α [57]. In the quiescent state, the regulatory domain of each kinase physically interacts with the cognate kinase domain, preventing catalytic activity. Binding to their upstream G-protein activators RAS and RHO, respectively, relieves these auto-inhibitory interactions, and both kinases are activated by acquiring an open conformation. In this conformation, the RAF1 regulatory domain can interact with the kinase domain of ROK-α and inhibit it. This physical cross-binding prevents the activation of ROK-α kinase activity and was the first demonstration that kinases can cross regulate each other in *trans* without intermediate steps or need for catalytic activity. Importantly, ROK-α does not seem to be regulated by BRAF. Ablation of *BRAF* in RAS-driven tumours did not result in a hyperactivation of ROK-α, indicating that both RAF isoforms play different roles in RAS mutant tumours [58]. The interaction between RAF1 and ROK-α may also be related to the anti-apoptotic signal mediated by RAF1, as activation of the FAS death receptor stimulates the formation of RAF1–ROK-α complexes [59]. This FAS-dependent induction of the RAF1–ROK-α complex seems to increase the threshold to trigger apoptosis upon FAS activation, and *RAF1* knock-out animals are hypersensitive to the induction of hepatocyte apoptosis by FAS. It seems that in foetal liver, the RAF1–ROK-α complex decreases the expression of FAS in the membrane. When the RAF1 inhibitory effect is lost, hyperactivation of ROK-α promotes the localization and clustering of FAS in the membrane, probably by reducing the internalization of this receptor, thereby decreasing the threshold of FAS sensitivity in this tissue.

### 2.3. Raf Kinase-Independent Regulation of Cell Cycle and Mitosis Checkpoints

**Figure 7:**
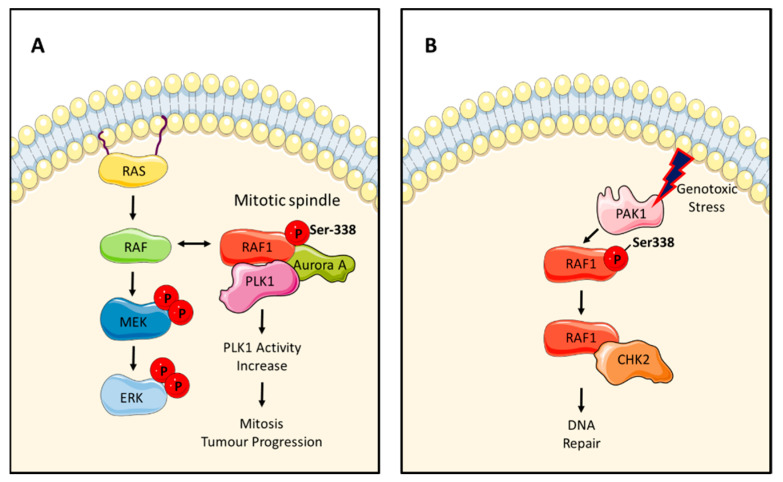
RAF1 kinase-independent regulation of cell cycle and mitosis check points. (**A**) RAF1 binds to PLK1 and Aurora A in the mitotic spindle and activates these kinases. (**B**) PAK1 promotes the interaction of RAF1 with CHK2 upon genotoxic effect, promotes DNA repair and prevents the activation of the DNA damage apoptotic pathway.

RAF proteins can drive cell cycle progression through the ERK pathway [5]. In recent years, accumulating evidence has suggested that RAF can regulate the cell cycle also in a kinase-independent fashion. One such a mechanism is mediated by the interaction between RAF1 and Polo-Like Kinase 1 (PLK1) and Aurora kinase A (Aurora A) [60]. These kinases are important regulators of mitotic progression and localize to the spindle poles and centrosomes during mitosis [61]. Cheresh’s group demonstrated that RAF1 regulates PLK1 and Aurora A in a kinase-independent fashion (Figure 7A). This work confirmed their previous observation that phosphorylation of RAF1 at Ser338 promotes the interaction of RAF1 with some of its kinase-independent effectors (Figure 3) and results in RAF1 binding to PLK1 and Aurora A at the mitotic spindle. This effect is specific of RAF1, since BRAF does not associate with PLK1 and Aurora A. Unlike the inhibitory interactions of RAF1 with MST2, ASK1 and ROK-α, the interaction of RAF1 with PLK1 promotes the activation of PLK1 kinase activity. In fact, expression of a phospho-mimetic kinase dead mutant RAF1-Asp338/Met375 promotes PLK1 activation and the progression of apoptosis. Importantly, this work showed that Ser338-phosphorylated RAF1 localised to the mitotic spindle in tumour samples. This was further supported by the identification of an allosteric small molecule inhibitor of RAF1, named KG5, that prevents the phosphorylation of RAF1 Ser338 and the activation of PLK1, causing mitotic arrest in prometaphase. This work first indicated that targeting RAF1 kinase-independent functions with small molecules is feasible and could be a new avenue for cancer treatment. 

The same group also described another kinase-independent function of RAF1 in cell cycle progression through regulation of Checkpoint kinase 2 (CHK2). CHK2 is a Ser/Thr kinase that is involved in the DNA damage response, cell cycle checkpoints, and activation of apoptosis [62]. The formation of the RAF1–CHK2 complex is regulated by PAK1 [63]. RAF1 binding to CHK2 promotes DNA repair and protects the cell from DNA damage (Figure 7B). This work showed that RAF1 Ser338 phosphorylation, but not RAF1 kinase activity, is necessary to mediate this effect in cells and xenograft tumours treated with ionizing radiation (Figure 3). In fact, phosphorylation of Ser338 is associated with radiation resistance, an increase in RAF1–CHK2 interaction, and CHK2 activation. This activation of CHK2 requires the phosphorylation of CHK2 Thr68 by another kinase. Importantly, the authors showed that treatment with KG5 prevents the phosphorylation of RAF1 at S338 and sensitizes the cells to radiation. These results further support the idea that targeting kinase-independent functions of RAF1 open new avenues for anticancer therapy, e.g., by enhancing apoptosis inflicted by DNA damaging agents. Despite the headlines made by targeted therapies, DNA damaging chemotherapy is still the mainstay of cancer treatment and augmenting its efficacy could reduce side effects and increase responses [64].

## 3. RAF Kinase-Independent Functions and KRAS Mediated Cancer: Opportunities for New Drug Targets

Treatment of RAS-mutated cancer remains one of the most urgent unmet needs in oncology. Despite the recent development of KRAS^G12C^ specific inhibitors that showed encouraging activity in clinical trials for lung cancer treatment, we still lack efficient treatments for most patients with RAS-mutated cancers [65]. Efforts to find RAS inhibitors proved futile over the last three decades, establishing the idea that RAS proteins are undruggable and that we should move the focus to targeting the main RAS effector pathways involved in oncogenesis, such as the ERK and the AKT pathways. While some of these strategies have shown positive results and have advanced to the clinic, most of them have shown limited efficacy and are not used as single agent therapies for the treatment of any cancer type [6,66]. 

An example are RAF inhibitors. They were designed to prevent the RAF kinase-dependent hyperactivation of the ERK pathway in RAS-mutated cancers, which is considered a main effector pathway of oncogenic RAS [5,6]. Highly potent RAF kinase-inhibitors were developed, which are effective in blocking signalling by BRAF^V600E^, but surprisingly induce a paradoxical activation of the ERK pathway in RAS mutant cells [9,10,11]. This is due to the induction of RAF dimerization as discussed above in Section 1. Three strategies have been tried to address this dilemma. The first was to combine RAF with MEK inhibitors to exert a “double block”. This approach was effective in BRAF^V600E^-mutated melanoma and is now a standard clinical treatment [67]. However, this combination is ineffective in RAS-mutated cancers including NRAS-mutated melanoma. The reason is that the topology of the ERK pathway combines a signal amplifier, i.e., the RAF-MEK-ERK kinase cascade, with a negative feedback from ERK to RAF. This constellation of a negative feedback amplifier makes a system robust against perturbation of the amplifier, i.e., MEK inhibitors, as the weakening of the negative feedback keeps the output constant despite the reduction in signal amplification [68]. To overcome this buffering requires inhibition of the input, i.e., RAF, but RAF dimerization and resistance of the dimer to RAF inhibitors reduce the efficacy of this approach. The second strategy was to design “paradox-breaking” RAF inhibitors, which do not promote dimerization and avoid the paradoxical stimulation of the ERK pathway [14,15]. However, these inhibitors also showed only marginal efficacy against RAS mutant tumours in animal models [14] and in clinical trials [69]. The reason is unknown but could be related to the observation that these inhibitors do not efficiently block the binding of RAF to RAS [70], which then could result in the formation of RAS-induced kinase-active RAF dimers. The third strategy was to exploit the fact that RAF dimers are structurally asymmetric, and that these differences in protein conformation between the protomers can dramatically reduce the affinity drug to the second protomer once it has bound the first protomer [13]. The reason for this can be explained by thermodynamic principles [71]. Indeed, using these principles to design a computational model of drug inhibition of RAF dimers considering structural features, phosphorylation, network context and genetic mutations enabled the identification of RAF inhibitor combinations that efficiently block signalling by mutant BRAF and mutant RAS [12]. Combining two structurally different RAF inhibitors that both bind to the ATP binding pocket seems counterintuitive. However, due to the slightly different conformations of the RAF protomers, each inhibitor only has high affinity for one protomer without competing for binding to the other protomer. This solution takes advantage of the large number of RAF inhibitors available and is equally efficient for blocking transformation by both BRAF and RAS oncogenes [12].

As the focus of drug development was on blocking the catalytic activity of RAF kinases, some of the clinical shortcomings of RAF inhibitors also may be due to the non-catalytic effects of RAF kinases that are likely to be differently affected by these drugs. For instance, we do not know whether and how current RAF kinase-inhibitors affect RAF1’s antiapoptotic kinase-independent functions. This is becoming important as current drug development is shifting from BRAF selective to pan-RAF inhibitors in the hope to block BRAF-RAF1 heterodimer signalling [8]. However, it will be equally important to assess how such inhibitors impact on the kinase-independent functions of RAF1 due to allosteric changes in protein conformation that could influence binding to ASK1 or MST2. This is emphasized by recent results from Barbacid’s group [50]. These works have focussed on the effect that *RAF1* ablation has in the development of murine lung adenocarcinomas induced by *KRAS* and *p53* mutations. Expression of KRAS^G12V^ in murine lungs resulted in the development of tumours, which was significantly reduced when *RAF1* was knocked out as well. Interestingly, ablation of *BRAF* did not reproduce this tumour-protective effect, suggesting that it is due to a specific RAF1 function. The deletion of *RAF1* was well tolerated by the animals and also seemed to avoid the development of resistant mechanisms. Knocking out *RAF1* also strongly reduced tumour burden in animals with concomitant *KRAS* mutation and deletion of *p53*, which produces a very aggressive phenotype that is common in human tumours [72]. Interestingly, loss of RAF1 expression impaired tumour formation by stimulating apoptosis that is not dependent on ERK activity, suggesting that it is the loss of RAF1-mediated MST2 and ASK1 inhibition that triggers apoptosis and restrains tumour growth. In support of this conclusion, the conditional expression of the kinase-dead RAF1^D468A^ and RAF1^K375M^ mutants from the endogenous locus had limited impact on the formation of lung tumours in the *KRAS^G12V^/p53^−/−^* mice [50]. These results clearly showed that the inhibitory effect of RAF1 on mutant KRAS-driven lung tumour progression is due to the kinase-independent functions of RAF1. Furthermore, results obtained in human patient-derived xenograft models strongly suggest that this critical kinase-independent RAF1 function is the inhibition of ASK1 and MST2 activation. Downregulation of the expression of ASK1 or MST2 blocked the proapoptotic signal caused by the loss of expression of RAF1. 

The important role of RAF1 for KRAS-mediated transformation was further demonstrated in a mouse model of pancreatic ductal adenocarcinoma (PDAC) [73]. PDAC is the most lethal paradigm of RAS-driven cancers. More than 90% of PDACs have *KRAS* mutations and are remarkably resistant to therapy [74]. In the mouse model, PDACs are induced via a combination of KRAS^GV12^ expression and *p53* deletion. In this model, ablation of *RAF1* or *EGFR* caused a delay of the formation of PDAC, whereas the concomitant knock-out of both *RAF1* and *EGFR* genes completely suppressed tumour development. Importantly, the systemic deletion of *EGFR* or *RAF1* did not decrease ERK or AKT signalling, and only produced mild toxicities. This is in apparent contrast to the significant side effects of drugs that block the catalytic activities of EGFR and RAF kinases. Provocatively, this may indicate that removing both the non-catalytic and catalytic functions may be better tolerated and more effective than just blocking the kinase activity. Interestingly, this study [73] also showed that resistance to *RAF1* or *EGFR* ablation separates two different groups of PDAC tumours at the molecular level. Transcriptome analysis showed that the two tumour types differed in the expression of genes related to apoptosis, ERK, PI3K, MYC and other well-known signalling networks. The relevance for human tumorigenesis was tested in xenograft models, where the concomitant ablation of *RAF1* and *EGFR* strongly suppressed PDAC formation. Intriguingly, none of the three RAF1 inhibitors tested showed any significant effect in these PDAC models, whereas *RAF1* ablation combined with treatment with the EGFR inhibitor gefitinib triggered cell death in several of the PDXs. These results further support the idea that the inhibition of RAF1 kinase-independent functions in combination with the catalytic inhibition of the EGFR might be an effective therapeutic strategy for the treatment of some PDAC patients. 

Taken together, these two studies clearly indicated that the effects shown in these models were due to kinase-independent signalling regulated by RAF1 that are related to the control of MST2 and ASK1 activation. Interestingly, these effects seem specific to RAF1 and could not be reproduced by a *BRAF* knockout. However, it will be very interesting to test the effects of ARAF and the BRAFV^600E^ mutant in this context. ARAF avidly binds to MST2 and is a strong suppressor of MST2 proapoptotic signalling [41]. Although wildtype BRAF does not bind to and regulate MST2, the BRAF^V600E^ mutant was shown to bind to and suppress activation of the closely related MST1 homologue in thyroid cancer [42]. 

## 4. Discussion

Thus far, the focus on blocking the RAS signalling effector was for inhibiting ERK activation by blocking RAF or MEK catalytic activities. However, recent data strongly suggest that we could find promising new drug targets by looking beyond the catalytic horizon. RAF kinases, as discussed above, have important functions which are independent of catalytic activities. Looking at the kinase-independent function of kinases may appear counterintuitive at first glimpse. However, consider that kinases are genuinely rather promiscuous enzymes which need to be targeted to substrates to achieve specificity [75]. Such targeting is usually mediated by protein–protein interaction (PPI) domains in the kinase itself or by scaffolding proteins that bind both the kinase and its substrate, thus forcing a specific interaction [76]. The RAF kinases use both themes. 

There is an abundance of scaffolding proteins that target RAF kinases to specific subcellular localizations and specific biological functions [76,77]. Importantly, they seem to dictate the context in which RAF paralog function. For instance, the RASSF1A tumour suppressor protein can disrupt RAF1–MST2 complexes, relieving the inhibition of MST2 and allowing MST2 to induce apoptosis [33]. Unfortunately, RASSF1A expression is often downregulated in cancer [78,79]. Conceptually, a drug that mimics the RASSF1A function of disrupting RAF1–MST2 interaction should have the same effect as expression of the RASSF1A tumour suppressor protein. As RASSF1A is downregulated in >80% of human cancers [78,79], this strategy could have wide applicability beyond RAS-driven cancers. 

This alone calls for a closer evaluation of the catalytic function independent effects of RAF kinases. An interesting aspect is that evolutionary BRAF is the oldest RAF isoform, while RAF1 and ARAF have been acquired more recently [80]. In terms of MEK kinase activity, BRAF is the most effective followed by RAF1, while the MEK kinase activity of ARAF is hardly measurable [81]. However, the complexity of regulation is inversely correlated with MEK kinase activity. As far as we know, BRAF features the simplest regulation, while RAF1 is intermediate, and ARAF regulation is most complex [5]. These observations suggest that much of the regulation is not about catalytic function but may be about (MEK) kinase-independent functions. This hint from evolution indicates that either undiscovered RAF substrates besides MEK exist that mediate tumorigenesis, or that RAF kinases have effector pathways that do not involve RAF kinase activity. As there is little evidence for alternative RAF substrates in the literature, focussing on RAF kinase-independent functions seems appropriate. Here, the targetable functions are to uncouple the control of RAF by disrupting the association between RAF and known effectors, such as MST2 and ASK1. This may be difficult given that PPIs are not easy to target. Alternatively, one may directly modulate the activity of RAF-controlled proteins. This will involve the design of kinase activators, e.g., for MST2 and ASK1. Although the usual strategy is to develop kinase-inhibitors, pharmacological kinase activators have been described, e.g., for AMPK [82], and may serve as useful leads. 

In summary, we are looking at an exciting new horizon of drug target discovery and development of RAS inhibitors based on mechanistic findings and network analysis. 

## Data Availability

Not applicable.
